# Algal Cells-Derived Extracellular Vesicles: A Review With Special Emphasis on Their Antimicrobial Effects

**DOI:** 10.3389/fmicb.2021.785716

**Published:** 2021-12-23

**Authors:** Fereshteh Bayat, Alireza Afshar, Neda Baghban

**Affiliations:** ^1^Department of Plant Genetics and Production Engineering, College of Agriculture and Natural Resources, Persian Gulf University, Bushehr, Iran; ^2^The Persian Gulf Biomedical Sciences Research Institute, The Persian Gulf Marine Biotechnology Research Center, Bushehr University of Medical Sciences, Bushehr, Iran

**Keywords:** exosome, algae, stem cell, antimicrobial, marine

## Abstract

Extracellular vesicles (EVs) originated from different cells of approximately all kinds of organisms, recently got more attention because of their potential in the treatment of diseases and reconstructive medicine. To date, lots of studies have been performed on mammalian-derived vesicles, but little attention has been paid to algae and marine cells as valuable sources of EVs. Proving the promising role of EVs in medicine requires sufficient resources to produce qualified microvesicles. Algae, same as its other sister groups, such as plants, have stem cells and stem cell niches. Previous studies showed the EVs in plants and marine cells. So, this study was set out to talk about algal extracellular vesicles. EVs play a major role in cell-to-cell communication to convey molecules, such as RNA/DNA, metabolites, proteins, and lipids within. The components of EVs depends on the origin of the primitive cells or tissues and the isolation method. Sufficient resources are needed to produce high-quality, stable, and compatible EVs as a drug or drug delivery system. Plant stem cells have great potential as a new controllable resource for the production of EVs. The EVs secreted from stem cells can easily be extracted from the cell culture medium and evaluated for medicinal uses. In this review, the aim is to introduce algae stem cells as well as EVs derived from algal cells. In the following, the production of the EVs¸ the properties of EVs extracted from these sources and their antimicrobial effects will be discussed.

## Introduction

Algae have been recently interested by scientists from different aspects including their stem cells and extracellular vesicles (EVs). Unlike animals, which typically develop through division of small undifferentiated stem cells, algal development (apart from undifferentiated apical stem cells) often involves fully differentiated cells that have low capacity for division and then revert to a state where nuclear mitoses and cell division resume.

Most complex multicellular algae usually have well-defined meristems that generate a diversity of differentiated cell types and tissues. These tissues typically include outer layers with protective epidermal cells and cells adapted for photosynthesis and interior cell layers that have more structural, reproductive, or transport functions. These meristems are niches for stem cells (Lenhard and Laux, 2003; Dodueva et al., 2017; Warghat et al., 2018). Stem cells are small morphologically undifferentiated cells with large nuclear/cytoplasmic ratios and a scant and poorly differentiated cytoplasm. Their main properties are a potential to go through numerous cycles of cell division while maintaining their undifferentiated state (self-renewal) and the potential to differentiate into one or more differentiated cell types (potency or potential). During tissue damage, stem cells take a main part in the maintenance of cell homeostasis and cell recovery. These stem cells need to be reached nearby or far away target cells during embryonic development and regeneration of adult tissues (Dodueva et al., 2017; Warghat et al., 2018). This communication is possible through soluble agents, direct cell-to-cell contact through long, thin tubular appendages, such as the cytonemes and cilia, or secretion of EVs (Jill Harrison, 2017). Recently, researchers have focused on plant’s-derived EVs as a flexible and suitable alternative to mammalian’s EVs regarding the role of EVs in cell-to-cell communications and its impact on medicine. This study was set out to evaluate the EVs of the algal cells and their possible antimicrobial effects.

## Stem Cell in Plants and Algae

It has been accepted that within both animalia and plantae, there is a special space called stem cell niches where stem cells are located. Animal stem cells are classified based on their ability to generate either a wide or a restricted pool of descendant cell types (Terskikh et al., 2006). The zygotic cell is the only mammalian totipotent cell that generates all embryonic and extraembryonic cell types. During embryonic development, pluripotent stem cells give rise to embryonic germ layers but can no longer produce an entire embryo. Finally, multipotent stem cells can only yield a range of different cells belonging to a single tissue.

Laux (2003) defined plantae stem cells as those in which generated daughter cells can either maintain stem cells nature and generate new stem cells, or undergo differentiation, which can include apical cells in tip-growing plants as well as intercalary meristems located within or at the base of plant organs. The studies on the genetic labeling of stem cells revealed that the whole body of a mature plant descends from small groups of stem cells in their growing apices, which these stem cells are maintained by signals from other neighbor cells.

Activation of cell fate in plants is space-dependent. Every plant cell follows a developmental program, which is driven by the position of the cell concerning its surrounding, rather than by the lineage-based differentiation program seen in animals. For this reason, differentiated tissues in plants can regenerate a totipotent embryo or a callus. A set of events that follow cell division and differentiation performed by a stem cell and its daughter cells summarizes the three defining characteristics for a stem cell; self-renewal, possession of undifferentiated characteristics, and ability to differentiate into an array of specialized cells (Scheres, 2007). In terrestrial plant biology, stem cells are largely considered in the context of meristems.

Eukaryotic algae are a diverse polyphyletic assemblage assigned to the kingdoms Chromista, Plantae, and Protozoa (Guiry and Guiry, 2018). The algae have differences with land plants from the appearance to the reproduction process. However, due to the fact that algae and land plants are a sisters’ group, they have some similarities, for instance, the existence of meristem, cell wall, totipotent cells, and etc. (McCourt et al., 2004). Due to these similarities, being a sister group with algae, and the reports of few studies on algal stem cells and EVs, the land plants were considered as an example for comparison with the plantae kingdom in order to more explain algae function and physiology. Moreover, because the algae have evolved multiple times independently of animals and land plants, they are natural experiments by which to explore the most diverse modes of cellular totipotency and stem cell ontogenies; algal multicellular body plans originated multiple times within diverse classes of Chromista and Plantae. Three algal lineages stand out for their complex morphologies and high diversity: brown algae (class: Phaeophyceae, with over 2,000 species); red algae (phylum: Rhodophyta, with over 7,500 species); and green algae (subkingdom: Viridiplantae in part the remainder being land plants).

Most complex multicellular algae usually have well-defined meristems generating a diversity of differentiated cell types and tissues. These tissues typically include outer layers with protective epidermal cells and cells adapted for photosynthesis and interior cell layers that have more structural, reproductive, or transport functions. Rigid cell walls constrain algal and land plant cells, including their stem cells, obscuring their functional homology with animal stem cells. Nevertheless, many of the properties of animal stem cells are also found in terrestrial plants, e.g., those associated with root and shoot apical meristems (Laux, 2003; Ivanov, 2007; Dodueva et al., 2017; Warghat et al., 2018), as it could be in the multicellular algae.

In multicellular algae, ontogeny generally can follow one of two developmental patterns: diffuse growth in which cell divisions can occur more or less throughout tissues of the organism, or division of dedicated stem cells, either solitary or in meristems, mostly apical, but sometimes intercalary. Diffuse growth, whether it occurs in multicellular filaments (e.g., the water silk *Spirogyra*) or multicellular sheets (e.g., the sea lettuce *Ulva*), results in little cell diversity and no identifiable set-aside cells, although the cells demonstrate virtual totipotency that is revealed through regeneration of a new thallus from thallus fragments or artificially created protoplasts.

It is not surprising that with the diversity of body plans, brown algae have a corresponding diversity of apical systems. Apical growth is considered ancestral in the class, as it is in land plants (bryophyte and vascular plants) and related green algae (Jill Harrison, 2017) and is typically generated by a single prominent apical cell at the apex of a filament or blade (e.g., *Dictyota*) or a band of apical cells at a blade apex (e.g., *Syringoderma* and *Padina*). In fucoids, brown algae, this apical cell is maintained in an apical pit and cuts-off derivatives from a mostly three-sided apical cell analogous to that in primitive mosses and liverworts (Renzaglia et al., 2018), which gives these algae the ability to generate three-dimensional forms like those of land plants. The convergent evolution of fucoid and land plant apical systems results in similar regular patterns (phyllotaxy) of lateral-branch or lateral-organ placement around the main axis of the plant body below the central apical cell or meristem that conforms to the Fibonacci series (Peaucelle and Couder, 2016).

Brown algae have two kinds of intercalary meristems: (1) trichothallic meristems in which cell division occurs at the base of a multicellular hair to produce filamentous or syntagmatic thalli and (2) more elaborate meristems that give rise to parenchymatous systems in kelp, another type of brown algae (Kawai and Henry, 2017). The intercalary meristems in kelp (i.e., in the order Laminariales) are analogous to certain types of terrestrial plant meristems. With elaborate differentiation yielding multiple cell types including outer epidermal cells, photosynthetic cells, and interior structural and transport cells, kelps resemble vascular plants in their complexity of cells and tissues. In most kelp species, individual plants consist of a stipe or stem-like organ that supports a blade, a flattened leaf-like organ. An intercalary multicellular meristem region of stem cells at the junction of these organs produces the cells required for elongation of both the stipe and the blade. These meristems can remain active for years. Perennial temperate to arctic species can exhibit seasonal growth cycles in which the blades detach above the meristem and a new one is regenerated *de novo* (e.g., *Laminaria hyperborea*). The kelp intercalary meristem is analogous to the vascular cambium (an interior ring of stem cells found in the stems of vascular plants), where cell division on one side of the ring produces cells that differentiate into water-transporting xylem and on the other side into photosynthate-transporting phloem, although functionally, they most resemble the intercalary meristem at the base of hornwort sporophytes or at the base of grass leaves.

Plant biologists recognize that protoplasts could take a prominent role in plant and algal cell totipotency, so it has a noticeable impact on algae and plant biotechnology (Reddy et al., 2008; Baweja et al., 2009; Baweja and Sahoo, 2009). These wall-less cells generate artificial stem cells that can be used on other cells, or used to induce somatic cell embryo formation (plant cloning), hybridize somatic cells, and genetically transform cells. The totipotency of protoplasts obtained from red, green, and brown multicellular algae and has been evaluated in culture (Kevekordes et al., 1993; Reddy et al., 2006; Fukui et al., 2014). Therefore, it can be concluded that even differentiated cells can return to stem cells with full totipotency if their cell walls and adjacent cells are removed.

## Plants and Algal Stem Cell’s Ability to Excrete Extracellular Vesicles

Today, the presence of vesicles has been proven in most prokaryotic and eukaryotic organisms. The nano-sized membrane vesicles are secreted from different cells of these organisms and released into the extracellular environment (Brown et al., 2015).

During tissue damage, stem cells take a main part in the maintenance of cell homeostasis and cell recovery. These cells could be detected using immune-labeling *via* EdU staining. The EdU staining could detect proliferative cells which are one of the characteristics of stem cells. Using this method, the proliferative/stem cells could be isolated and used for further analysis (Hong et al., 2015). Moreover, these stem cells need to be reached nearby or far away target cells during embryonic development and regeneration of adult tissues. This communication is possible through soluble agents, direct cell-to-cell contact through long, thin tubular appendages, such as the cytonemes and cilia, or secretion of extracellular vesicles EVs (EVs). These vesicles with 30–3000 nm in diameters have free diffusion factor properties and a wide cell membrane and cytoplasmic organization. They have distinct biological compositions depending on size and origin and hence, their functions maybe vary (Aliotta et al., 2012; Camussi et al., 2013).

The composition of EVs consists of different molecules, which important components are metabolites, proteins, nucleic acids, and lipids (Table 1). The cargo of EVs is mainly dependent on the nature and origin of the primitive cells or tissues and the isolation technique (Kolonics et al., 2020; He et al., 2021). Some of these techniques are ultracentrifugation and chemical precipitation method *via* commercial EVs kit that both are very common in use (Afshar et al., 2021; Zhankina et al., 2021). The maintenance of tissue homeostasis is regarded as one of the most important functions of EVs. There is a mutual interaction between EVs secreted from damaged cells and stem cells, as EVs secreted from injured tissue affect stem cells, reciprocally splashed EVs of stem cells support injured tissue. Hence, the EVs extraction and purification methods highly affect EVs characteristics, the International Society for Extracellular Vesicles (ISEV) has determined standards of EVs purification (Théry et al., 2018; Russell et al., 2019).

**Table 1:** The components of EVs(EV) and their biological function (Alfieri et al., 2021).

Component		Biological function
Lipids	Sphingolipids	The high enrichment of GIPCs in plant EVs is suggestive of a signaling function of the EV membrane, especially in the extracellular ROS burst, as proven in Arabidopsis plants
	Glycosylinositolphosphoceramides (GIPCs)	
	phosphatidylethanolamine(PE)	PA is as an important class of lipid messengers involved in many cellular processes such as cytoskeletal organization, cell proliferation, and survival
	phosphatidylcholine (PC)	
	phosphatidylinositol (PI)	
	and phosphatidic acid (PA)	
Proteins	cytosolic proteins (e.g., actin and proteolysis enzymes)	vesicle stability in the case of plasma membrane vesicles purified from broccoli plants
	membrane channel/transporters (e.g., aquaporin and chloride channels)	
	Aquaporin	
	different hydrolases (ATPases, pectinesterase, phospholipases, amylases, _ galactosidases, and adenosylhomocystein hydrolyse),	
	enzymes (SODs, CATs, PODs, and GPXs)	
Nucleic Acids	mRNA, miRNA, DNA	play a role in inter-kingdom communication
Plant Metabolites	carbohydrates (glucose, fructose, sucrose)	Cell homeostasis
	amino acids (alanine, asparagine isoleucine, threonine, leucine)	
	organic acids (mainly glycolic and citric acids),	
	sugars and sugar derivatives	
	bioactive compounds, such as quinic acid, myo-inositol, and aucubin	

As revealed so far, approximately all cells of different organisms can secrete EVs, although they might be different types, pose different functions depending on origin. Therefore, choosing the ideal cells to get EVs with the desired function needs to be concerned. Here, one of the most important sources of EV extraction and its therapeutic effects explain as an example of the therapeutic effects of EVs. The recent studies on mesenchymal stem cells (MSCs) revealed that they can be an effective branch of stem cells in therapeutic applications (Cui et al., 2018; Zhao et al., 2018; Riazifar et al., 2019). Exosomes derived from MSCs have either the advantages of exosomes, or the characteristics of MSCs, and their therapeutic effects have been proved in different diseases in recent studies (Bolivar-Telleria et al., 2018; Moon et al., 2019).

To use the advantages of exosomes in therapeutics, the optimized purification method to get a large amount of non-toxic homogenized exosomes, as well as efficient transfection strategies, is needed (Kooijmans et al., 2012; Yamashita et al., 2018).

Plant-derived exosomes, as one of the sister groups of algae, recently get great attention as a suitable alternative to mammal’s exosomes, because of their physiological, chemical, and biological characteristics, which make them a proper candidate to cope with the technical limitations of mammalian vesicles. Regente et al. in 2009 reported the presence of exosome-like vesicles with 50–200 nm in diameter in sunflower seeds (Regente et al., 2009). Far along, the isolation of vesicles by ultracentrifugation from different plant species like grape, grapefruit, ginger, and broccoli (Ju et al., 2013; Wang et al., 2014; Zhuang et al., 2015; Deng et al., 2017) has been reported that allows their effective and abundant production.

Facile large-scale production (Li et al., 2018), low toxicity, reduced immunogenicity (Deng et al., 2017), efficient cellular uptake (Wang et al., 2013), and high biocompatibility and stability (Zhang et al., 2016) make plant-derived EVs as promising therapeutic factors or drug deliver nanoparticles in medical applications in compared with MDEs or artificial nanoparticles.

Despite lots of studies on the bioactive content of the plant EVs, still, further studies are necessary to understand the bioactivities and applications of plant EVs. Besides plant-derived EVs, our knowledge on marine cell-derived EVs remains extremely limited, while they can be a more accessible source to produce a large amount of EVs very fast and easily. Algae as an important marine source for EVs are very economical compared to edible plants and can be grown in any place to get EVs within about 1 week, therefore can establish facile scaled-up production of pure EVs with high quality (Kuruvinashetti et al., 2020).

Therapeutic applications of EVs, in addition to their content, depend on their capability to cross barriers like the cytoplasmic membrane and blood/brain barrier. In mammalian and plant EVs, the mechanism of absorption is different, or they are absorbed either through endocytosis or through the fusion of vesicles and plasma membranes (Rome, 2019). Therefore, membrane properties of exosomes play an important role in crossing cellular barriers. In algae derived EVs, where the membrane is rich in beta proteins, the membranes are easier to attach. Thus, along with biocompatibility, no toxic effect on cells/tissues and organs, nano-nature, increasing circulatory stability, and low immunogenicity make algae a sustainable marine source for the production of exosomes for their potential use in medical and therapeutic applications (Kuruvinashetti et al., 2020).

## The Antimicrobial Effects of Plant’s Evs

**Figure 1:**
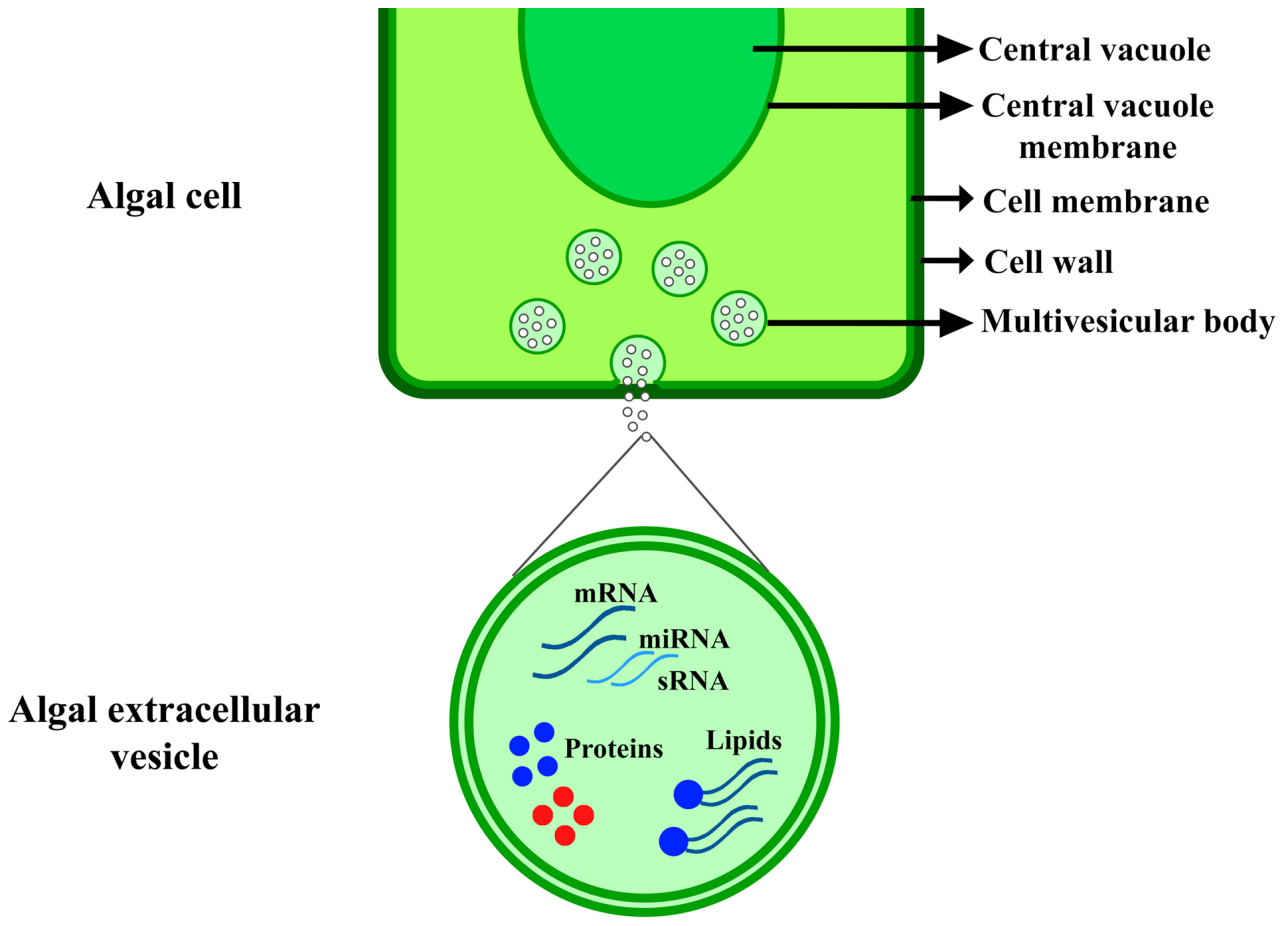
EVs production in algal cell. The extracellular vesicle includes nucleic acids, such as mRNA, microRNA and small RNA (sRNA), proteins, and lipids, which were explained in Table 1.

Plant-derived EVs because of their biological characteristics got more attention in recent years, many studies emphasize their role in the immune response against invading pathogens (Rybak and Robatzek, 2019; Kolonics et al., 2020). Actually, involving EVs in pathogenesis is two-sided, and some pathogens like bacteria, fungi, and parasites also depend on EVs cargo to exploit their host (Kuipers et al., 2018; Liu et al., 2018; Bielska et al., 2019; Ofir-Birin and Regev-Rudzki, 2019). Therefore, it is accepted that Evs have a key role in plant-pathogen interactions and many studies have been proved it (Figure 1; Boevink, 2017; Hansen and Nielsen, 2017; Rutter and Innes, 2018). The first evidence of antimicrobial nature of plant-derived EVs was showed in barley against powdery mildew fungus *Blumeria graminis* (An et al., 2006), later in sunflower against phytopathogenic fungus, *Sclerotinia sclerotiorum* (Regente et al., 2017), and in Arabidopsis against bacterial plant pathogen, *Pseudomonas syringae* (Rutter and Innes, 2016).

EVs involving in plant-pathogen interactions as well as the proteome of EV derived from uninfected Arabidopsis rosettes and apoplastic fluids pathogen-infected have been recently analyzed (Rutter and Innes, 2016). EVs derived from extracellular fluids of tomato (De Palma et al., 2020) and sunflower seedlings (Regente et al., 2017) as well as those derived from leaf apoplastic fluid of *N. tabacum* and *C. plantagineum* (Woith et al., 2021) have been known to be involved in plant-microbe interactions. In most cases, EV secretion increases by pathogen invading and raises the severe role of EV in plant defense mechanisms. The proteome analysis of EVs derived from these mentioned plants showed that these EVs are enriched in proteins involved in signal transmission in response to biotic and abiotic stresses, immunity responsible proteins, cell wall remodeling enzymes as well as a protein involved in plant-microbe interactions. Table 2 lists some of these proteins raised from proteome analysis of plant-derived exosomes.

**Table 2:** Proteins list from proteome analysis of some plant-derived exosomes.

Plant	Description	References
*N. tabacum* and *C. plantagineum*	annexin D5-like	Woith et al., 2021
	clathrin heavy chain 1-like	
	coatomer subunit alpha-1-like	
	coatomer subunit beta-1	
	coatomer subunit beta’-2-like	
	coatomer subunit gamma	
	patellin-3-like isoform X2	
	tetraspanin-3-like	
	tetraspanin-8-like	
	endochitinase EP3-like	
	G-type lectin S-receptor-like serine/threonine-protein kinase At1g34300	
Arabidopsis	RABD2a/ARA5 (Golgi/TGN/EE/secretory vesicles)	Rutter and Innes, 2016
	Plasmodesmata	
	RABG3f (LE/MVB/tonoplast)	
	RABF1/ARA6 (LE/MVB)	
	PM	
	CLC2 (clathrin-coated vesicle pits)	
	GOT1 (Golgi)	
	VAMP711 (tonoplast)	
Tomato	endochitinase	De Palma et al., 2020
	patatin-like protein 2	
	glucan endo-1,3-beta-glucosidase B precursor	
	hypersensitive-induced response protein 1	
	calmodulin 5,460,408,499 trypsin inhibitor 1-like	
	probable linoleate 9S-lipoxygenase 5	
	annexin p34	
	lysM domain-containing GPI-anchored protein 2	
	ethylene-responsive proteinase inhibitor 1	
	putative late blight resistance protein homolog R1A-10	
	putative late blight resistance protein homolog R1A-3	
	NDR1/HIN1-like protein 3-like isoform X2	
	putative LRR receptor-like serine/threonine-protein kinase At4g00960	
	putative late blight resistance protein homolog R1A-3	
	basic 30 kDa endochitinase	
	germin-like protein subfamily 1 member 19	
	CASP-like protein PIMP1	
	probable LRR receptor-like serine/threonine-protein kinase At1g06840	
	hypersensitive-induced response protein 1	
	monocopper oxidase-like protein SKU5	
	wound/stress protein precursor	
	MRLK1 serine/threonine-protein kinase, partial	

The proteome analysis is still in the early days and to get a clear clue large amount of replication, precise, and continuous processing of high-quality data and reference genomes are needed; however, the results to date provide candidate logical components in the interactions between plants and pathogens.

**Figure 2:**
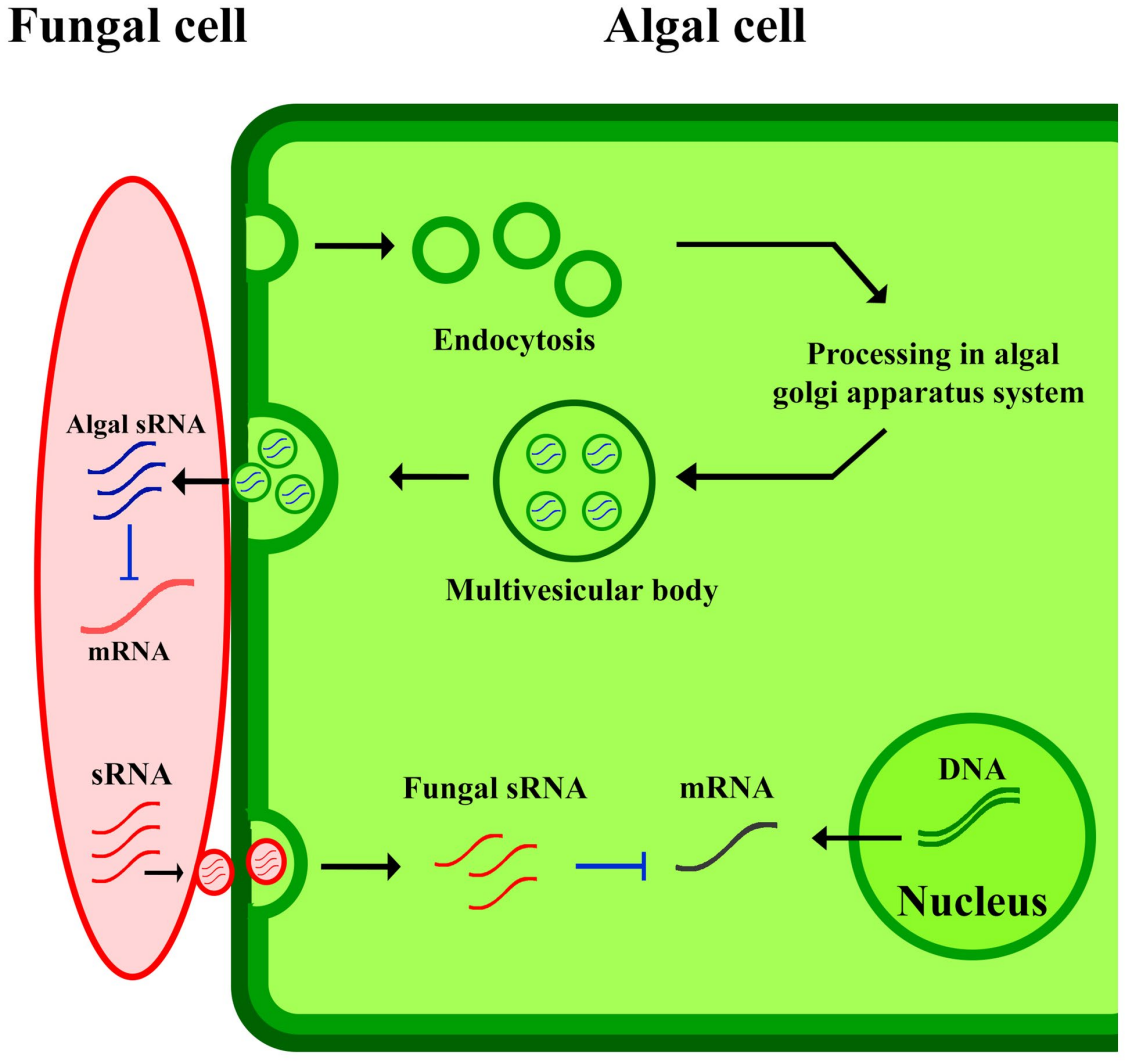
EVs-pathogen interaction in plants immunity responses. The algal sRNA in EVs suppressed fungal mRNA production in algal-fungal interaction.

Cross-kingdom RNA interference can be explained as one of the possible mechanisms involved in plant-derived EVs-pathogen interaction in plants immunity responses (Figure 2). The study of the gray mold caused by *Botrytis cinerea* in *A. thaliana* and *Solanum lycopersicum*, revealed small RNAs (sRNAs) of *B. cinerea* where they were revealed to be transferred from the fungus to the host to silence plant immunity genes (Cai et al., 2019). In response to pathogens, plants deliver sRNAs into the fungus using exosomes to limit the virulence potential of the organism upon knockdown (Lu et al., 2018). These mechanisms are widespread in other pathogens infected plants, such as cotton plants infected by *Verticillium dahliae* (He et al., 2016), and wheat plants for suppressing the invasion of *Fusarium graminearum* (Jiao and Peng, 2018). At some point, additional studies will be needed to better explain the subsets of EVs involving in the transfer of sRNAs into invading pathogens. It appears probable that both EV-dependent and -independent mechanisms will be discovered for facilitating this transfer.

## The Antimicrobial Effects of Algal Evs

Microorganism’s communication in the marine environment has a great impact on trophic level interactions and population substitution, awareness of EVs importance in cell-to-cell communication raises the question of how EVs participate in these processes. Early evidence from marine EVs came from model alga *Emiliania huxleyi*, studies demonstrated that vesicles generated over viral infection by this organism act as a pro-viral signal, through accelerating infection and increasing the half-life of the virus in the extracellular milieu (Schatz et al., 2021). Later authors profiled the sRNA cargo of vesicles generated by *E. huxleyi* over bloom succession and concluded that *E. huxleyi*-derived vesicles modulate host-virus dynamics and other components of the microbial food webs, so highlighting the importance of EVs to microbial interactions in the marine environment.

Generally, plant-derived vesicles reveal a broad therapeutic potential, which can help patients, and may establish the future generation of therapeutics.

## Future Aspect of Antimicrobial Products of Plant Evs

To get clear insights on the exact role of EVs originated from different cells, more studies on their biological characteristics and interactions are needed. Studies on plants’ EVs revealed similar intrinsic therapeutic materials as mammal’s EVs, while there are some advantages on plants’ EVs compared to mammal’s EVs. First, they can be obtained from a variety of renewable sources; moreover, allowing researchers to select their desired EVs with precise effects on disease, also facilitates its large-scale production. Second, EVs’ component seems to be evolved naturally in plant cells which makes them biocompatible and non-toxic. The EVs’ lipid membrane stability helps them to be simply adapted to target specific ligands, gives them the potential use as drug delivery nanocarriers. Moreover, plant-derived vesicles can be examined in a comparably short time through eco-friendly protocols (Zhang et al., 2016; Pocsfalvi et al., 2018; Wiklander et al., 2019). Besides these advantages, there are still some concerns on plant EVs to be solved. The standard isolation techniques with low cost and complexity and increase purity should be established for mass-production of high-quality exosomes for the use in therapeutic applications (Ludwig et al., 2019). Primary, the exact content and functionality of the miRNA, mRNA, proteins, and lipids in the exosomes have been unknown so far (Lee et al., 2012). Second, in spite of the developments in exosome isolation methods, a gold standard has not been yet presented (Ludwig et al., 2019). The isolation process cost and difficulty should be decreased, while the exosome purity should be enhanced. Third, mass-production of high-quality exosomes should be probable for the therapeutic applications.

A multi-functional system with a highly efficient isolation technique and real-time quantification and analysis technology is needed for efficient applications. Also, to keep EVs components, such as proteins and RNAs, storing below −70°C (Huang et al., 2020), or freeze-drying is recommended (Charoenviriyakul et al., 2018). Though, long-term preservation using these approaches is still not clarified to be applied in the diagnosis and therapeutic applications (Li et al., 2018). In addition, optimization of isolation approaches for should be performed to obtain uniform nanovesicles. Additionally, a detailed evaluation of their morphological features, the quantitative aspects, and chemical components should be performed to attain evidence on their functional roles. Lastly, exosomes might be the important element in the medicine in future.

## Author Contributions

All authors listed have made a substantial, direct, and intellectual contribution to the work, and approved it for publication.

## Conflict of Interest

The authors declare that the research was conducted in the absence of any commercial or financial relationships that could be construed as a potential conflict of interest.

## Publisher’s Note

All claims expressed in this article are solely those of the authors and do not necessarily represent those of their affiliated organizations, or those of the publisher, the editors and the reviewers. Any product that may be evaluated in this article, or claim that may be made by its manufacturer, is not guaranteed or endorsed by the publisher.
